# Kinetic and thermodynamic studies of eicosapentaenoic acid extraction from *Nannochloropsis oceanica* using tetramethyl ammonium chloride and microwave irradiation

**DOI:** 10.1371/journal.pone.0267626

**Published:** 2022-05-05

**Authors:** Shiva Rezaei Motlagh, Ramin Khezri, Razif Harun, Dayang Radiah Awang Biak, Siti Aslina Hussain, Ching Yern Chee, Soorathep Kheawhom

**Affiliations:** 1 Department of Chemical Engineering, Faculty of Engineering, Chulalongkorn University, Bangkok, Thailand; 2 Department of Chemical and Environmental Engineering, Faculty of Engineering, University Putra Malaysia, UPM, Serdang, Selangor, Malaysia; 3 Department of Chemical Engineering, Faculty of Engineering, University of Malaya, Kuala Lumpur, Malaysia; 4 Research Unit of Advanced Materials for Energy Storage, Chulalongkorn University, Bangkok, Thailand; 5 Bio-Circular-Green-Economy Technology & Engineering Center (BCGeTEC), Faculty of Engineering, Chulalongkorn University, Bangkok, Thailand; NOVA University Lisbon, PORTUGAL

## Abstract

Microalgae have garnered widespread attention as a sustainable source of pharmaceuticals and nutraceuticals. As for extracting lipids from microalgae, the combination of microwave-assisted extraction (MAE) and ionic liquids (IL) is shown to be promising. However, such an undertaking usually requires a large consumption of expensive ILs. This study innovatively employs tetramethyl ammonium chloride ([TMAm][Cl]) as an additive in water medium to associate with microwave-assisted ionic liquid extraction (MAILE) in extracting lipids from *Nannochloropsis oceanica* (*N*. *oceanica)* microalgae. In extraction, knowledge of reaction kinetics is crucial since it provides the foundation for developing, controlling, and improving the processes of extraction. Herein, using MAILE, lipids are extracted from *N*. *oceanica* microalgae and transesterified to eicosapentaenoic acid (EPA). Mass transfer kinetics are, therefore, investigated using the first and second-order rate law and Patricelli’s model. In the development of models, the influence of temperature (60–90°C) and reaction time (1–25 min) on EPA extraction is empirically evaluated. From the thermodynamic study, the positive values of *ΔS* (+0.10 kJ mol^-1^K^-1^) and *ΔH* (+32.50 kJ mol^-1^) and the negative value of *ΔG* (-1.68 to -4.75 kJ mol^-1^) confirm that this process is endothermic in nature, irreversible and spontaneous. MAILE proves to be a promising approach for the extraction of high-quality EPAs. Due to its low cost, rapid operation, and environmental friendliness, it is seen to be suitable for both pharmaceutical and nutraceutical applications.

## 1. Introduction

According to the food standard agency (FSA), the human body needs regular consumption of omega-3 polyunsaturated fatty acids (PUFAs), mainly in the form of eicosapentaenoic acid (EPA) [1, 2]. EPA consumption on a regular basis helps to diminish the risk of stroke, cardiovascular and neurological illnesses, rheumatoid arthritis, and other biological threats [3]. Microalgae are primary sources of EPA recognized as virtually essential omega-3 PUFAs [4]. *Nannochloropsis oceanica* (*N*. *oceanica*) is cultivated microalgae that has significant levels of EPA in its triglycerides and polar lipids [5]. The process of producing fatty acid methyl esters (FAMEs) from microalgal lipids entails multiple processes, including cultivation, harvesting, drying, lipid extraction, transesterification, and purification [6]. In the extraction of lipids from microalgae, cell rupture proves to be a complex process, particularly for *N*. *oceanica*, due to the microalgae’s thick cell wall. The amount of extracted lipids is governed by the level of the fragmentation cells, which is the rate-limiting phase in the process of extraction [7]. Meanwhile, during cell disruption, the microalgae’s cell wall prevents the solvent from accessing the cell’s intercellular contents and ruptures, thus releasing lipids [8].

ILs are organic salts that can provide superior features in cellulosic biomass treatment applications. Such a process includes thermal stability across a wide temperature range, having modifiable physicochemical properties and relatively low vapor pressure [9]. ILs have the potential to be used in a diverse range of applications, from industry to green chemistry. However, due to high cost and environmental concerns connected with their disposal, particularly in refining large-scale lignocellulosic biomass, ILs have their drawbacks but these can be resolved since they are highly recyclable [10].

ILs can enhance the yield of lipids recovered from algae owing to its high solubility in lignocellulosic materials, which are the principal components of the cell wall [11]. The impact of ILs on increasing the yield of lipids extracted from *Chlorella vulgaris microalgae has previously been* studied [12]. Thus, when 1-ethyl-3-methyl imidazolium acetate, 1-ethyl-3-methyl imidazolium diethylphosphate, 1-ethyl-3-methyl imidazolium tetrafluoroborate, and 1-ethyl-3-methyl imidazolium chlorides were used as solvents, the content of the extracted FAMEs was found to be greater than 200 mg g^-1^. Yet, when hexane-methanol was used, the yield only reached 185.4 mg g^-1^. Previous experiments have suffered not only from the use of large quantities of ILs but also from greater extraction temperatures (100–120°C) as well as longer time spans [12–14].

Most microalgae species have thick cell-walls, which are resistant during the stages of extraction. Therefore, one is required to use a cell disruption method such as mechanical pressing, exposure to pulsed electric fields, microwave irradiation, ultrasound, and supercritical fluid treatments [15]. MAE has been demonstrated to be an effective and straightforward method for extracting lipids from microalgae [16]. Yet, it may not be as effective in solutions having a high inherent resistance [17]. A few studies have been applied to evaluate kinetic models for the extraction of total lipids using MAE [18–20]. Such studies have focused on the extraction of bioactive compounds using phenol via MAE [21] and comptothecin from Nothapodytes via MAE [22]. In the extraction of compounds, the combination of microwave energy along with IL in MAILE has been shown to be promising in enhancing the rate of mass transfer. MAILE has further advantages, including shorter reaction time and solvent consumption [23, 24]. In this novel approach, applying precisely controlled temperature and pressure, and microwave irradiation ILs can improve mass transfer by improving solution conductivity. When these two approaches are combined, as in MAILE, higher compound extraction occurs in comparison to conventional extraction procedures [25, 26]. Besides, two fundamental phenomena occur: 1) water molecules inside the cell evaporate with the increased temperature generated by microwave irradiation. Hence, the cell walls of microalgae break, causing the plant matrix to enlarge and crack. 2) extracting components depart the cell and enter the solvent medium as a result of the mass transfer gradient induced by IL [27]. It is noted that microwave irradiation can be absorbed by organic components found in oil, allowing for easier extraction [18, 28]. The novelty of this work is mostly related to the design of the experiment. Controlling factors such as reaction time and temperature is crucial for optimizing the extraction process and, consequently, the economic viability of the technology [29].

The study of kinetics provide knowledge based on the rate of extraction: whether the process is slow/fast enough to reach equilibrium [30]. Such studies aid in comprehending the factors affecting extraction rate, which will be used to improve the whole process and plan for future scaling [31]. Reaction order is also important because it establishes a relationship between the concentration of the reactant and reaction rate [32]. Numerous kinetic models have been developed by mathematical methodologies to simulate the extraction kinetics of plants. Theoretically, models can be created using Fick’s law or chemical kinetic equations (rate law), or they can be derived empirically or semi-empirically [19].

Extraction kinetic models based on chemical kinetic equations or rate laws (first-order and second-order) have been applied to both conventional and non-conventional extractions; the latter is the most frequently used. To represent the variation of chemicals in extracted products, kinetic models may employ empirical equations; such equations provide little understanding of the processes of extraction [29]. Results of the models used can vary according to parameters applied, such as extraction target compounds, cellular structures associated with the various types of biomass, and processing conditions [19].

The extraction of essential oils from patchouli leaves (*Pogostemon cablin Benth*) using MAE was reported using first-order kinetic modeling [33]. Furthermore, second-order kinetic modelling was utilized to describe the MAE of chemicals extracted from Vernonia cinerea leaf [34], oil extraction from sandalwood [35], and the phenolics and antioxidants content of Turkish artichoke [36]. Patricelli’s model has been frequently used to examine MAE for lipid extraction from *Nannochloropsis sp*. [19], antioxidant phenolic compounds from Brewer’s Spent Grain [37] and essential oils from (*Citrus sinensis (L*.*) Osbeck*), pomelo (*Citrus grandis L*.), and lemongrass (*Cymbopogoncitratus*) [38].

Activation energy of extraction is a term used to describe the energy barrier that must be overcome in order to accomplish the process of extraction [39]. Therefore, a thermodynamic model was developed to determine the activation energy and the thermodynamic state of extraction using factors such as enthalpy, entropy, and free Gibbs energy [32]. A number of researchers have investigated the kinetics and thermodynamics of reactions involved in total lipid production via conventional extraction methods [40]. However, the principal mechanism that handles the mass transfer of intracellular lipids into the organic solvent for non-conventional techniques is not well explained. To date, no mathematical model has been developed to adequately describe the kinetics or thermodynamics of EPA extraction using MAILE.

Current research has found a unique extraction approach that allows for the use of an IL solution mediated in water in place of the more expensive pure ILs necessary for MAILE without significantly reducing the extraction yield of EPA. It is suggested that such an approach can help to overcome the problems that exist in attaining a large-scale and economic criterion. Thus, [TMAm][Cl] was chosen to screen ILs for the highest extraction capacity towards the extraction of EPA [41]. The extraction solution was prepared having a ratio of 3.3% w/v of IL to water; the method of preparation has been adapted from our previous study [42]. In this approach, a solution of microalgae, IL and water was heated by microwave irradiation to undertake the extraction of total lipids. Via the transesterification process, total lipids convert into fatty acids from which the produced EPAs are measured.

Herein, this paper investigates the influence of extraction temperatures (60–90°C) and reaction time (1–25 min) on the extraction of EPA. Kinetic models are developed based on first-order and second-order rate laws and Patricelli’s model [43]. Subsequently, the thermodynamic study of EPA extraction is implemented to determine Gibbs free energy, enthalpy, and entropy of the system. The aim of this research is to obtain high-quality lipids and EPAs for pharmaceutical and nutraceutical applications via a process that is faster, less expensive, and more environmentally friendly.

## 2. Materials and methods

### 2.1. Materials

The dried powder: *N*. *oceanica* microalgae batch no. (LYPH20180924) was bought from Xi’an Lyphar Biotech Co., LTD, Xian City, China. Hexane (C_6_H_14_, 95%), chloroform (CH_3_Cl_3_, >99.8%), methanol (CH_3_OH, 99.9%) and hydrochloric acid (HCl, 99%) were procured from R&M Chemicals, Malaysia. The IL i.e. tetramethyl ammonium chloride ([TMAm][Cl], ≥99%) was purchased from Sigma-Aldrich, Malaysia. All other chemicals and organic solvents were of analytical grade. To assure the precision of the outputs, experiments were replicated three times.

### 2.2. Microwave-assisted extraction of EPA using [TMAm][Cl]

**Fig 1:**
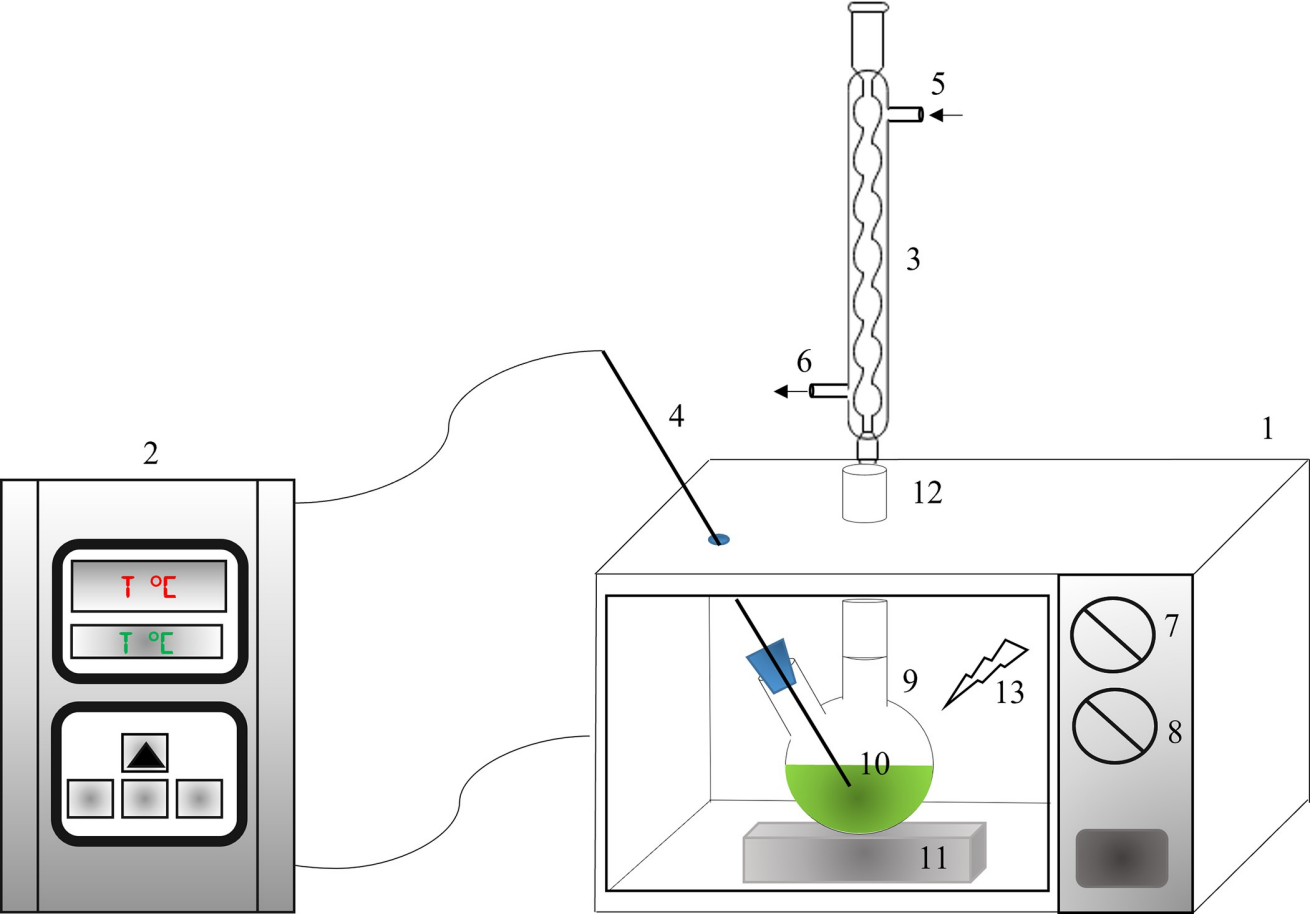
Schema of experimental setup for MAILE: (1) Modified microwave oven, (2) Signal transmitted controller with on/off relay switch, (3) Reflux condenser, (4) Thermocouple, (5) Cold water in, (6) Hot water out, (7) Power regulator, (8) Timer, (9) Round bottom flask, (10) Ionic liquid solution and algae powder, (11) Ceramic pad, (12) Connecting tube, and (13) Irradiation microwave zone.

Microwave irradiation was carried out via a domestic microwave oven (Samsung, ME711K, Samsung, Port Klang, Selangor, Malaysia) having an operating frequency of 2.45 GHz. In Fig 1, the MAILE experimental setup is depicted. The microwave oven was modified to accommodate the installation of a thermostat and condenser on top of the microwave body. A round-bottomed flask containing the microalgae sample was placed in the microwave oven, which is connected to a reflux condenser. A chiller was used to maintain the water temperature at 20°C, which was also used to condense the evaporated water in the condenser column during operation. A controller box was used to indicate the real time and to control temperature, which ranged from 20–240°C. Temperature control was accomplished using an auto-on/off switch for the microwave power, which can reach a maximum output of 800 W.

In our research, we inculcated optimum conditions from earlier work [42] using 0.5 g of dried *N*. *oceanica* with solid-loading of 1% w/v, which was mixed with 1.65 g of [TMAm][Cl] and added to the mixture. The solution was finally irradiated by microwave under the constant power of 700 W. Then, it was placed in the microwave subjected to different durations of exposure. Next, the microwave reactor was started, and heated at temperatures of 60 to 90°C and held for 1–25 min. Once microwave procedures were completed, a mixture of CHCl_3_/CH_3_OH (2:1, v/v) was added to the extraction product in order to dissolve the lipid content. The solution was separated into different layers via centrifugation at 4000 rpm for 5 min. The bottom layer containing the organic phase was then separated and washed 3 times with C_6_H_14_/H_2_O (1:1 v/v) to remove the polar compounds and residual ILs and left inside the fume hood for 3 to 4 days. Finally, once the hexane evaporated, crude lipids were obtained and determined gravimetrically.

### 2.3. Transesterification of lipids

The process of transesterification was performed by adding the mixture CHCl_3_/CH_3_OH/HCl (1:10:1, 3 ml) to the extracted total lipids and heated in the oven for 60 min at 90°C. The mixture was left to cool down to 25°C. A mixture of CHCl_3_/C_6_H_14_ (1:4 v/v, 3.3 ml) was added to the solution after it was diluted with 1 ml of distilled water. As a result, two separate layers formed in the solution. The so-called organic phase that contained the FAMEs was then collected from the top layer and underwent evaporation [44]. The total yield of FAMEs can be calculated as follows:

$$\mathrm{total}\;\mathrm{FAMEs}\;\mathrm{yield}\;(\mathrm{mg}/g)=\frac{\mathrm{mass}\;\mathrm{of}\;\mathrm{extracted}\;\mathrm{FAIMEs}\;(\mathrm{mg})}{\mathrm{mass}\;\mathrm{of}\;\mathrm{dried}\;\mathrm{microalgae}\;(g)}$$ (1)

A capillary column ZB-WAX (30 m × 0.32 mm × 0.2 *μ*m) was used to separate the FAMEs from the solution. As for the carrier gas, helium (2 ml/min) was used. Sample injection in split mode (10:1) was carried out. Samples were heated in the oven initially at 100°C for 1 min and then at 230°C for 5 min. Temperature was maintained at 250°C. A gas chromatography with flame ionization detector (GC-FID) was used for quantification and elemental analysis of FAME. In the analysis, the area of each peak corresponding to FAMEs was compared to the area of the standard FAMEs provided by Marine Oil FAME Mix (Restek Corp., Bellefonte, PA USA). In Eqs (2) and (3), the calculation of EPA recovery yield is explained in terms of percentage recovered (wt.%) and mass yield (mg g^-1^_FAMEs_), respectively:

$$\mathrm{EPA}\;\mathrm{percentage}\;(\%\;\mathrm{wt}.)=\frac{\mathrm{EPA}\;\mathrm{peak}\;\mathrm{area}}{\mathrm{total}\;\mathrm{area}}\times 100$$ (2)

$$\mathrm{EPA}\;\mathrm{content}\;(\mathrm{mg}/g)=\frac{\mathrm{total}\;\mathrm{FAMEs}\;(\mathrm{mg}/g)\times \mathrm{EPA}\;\mathrm{percentage}\;(\%\;\mathrm{wt}.)}{100}$$ (3)

**Fig 2:**
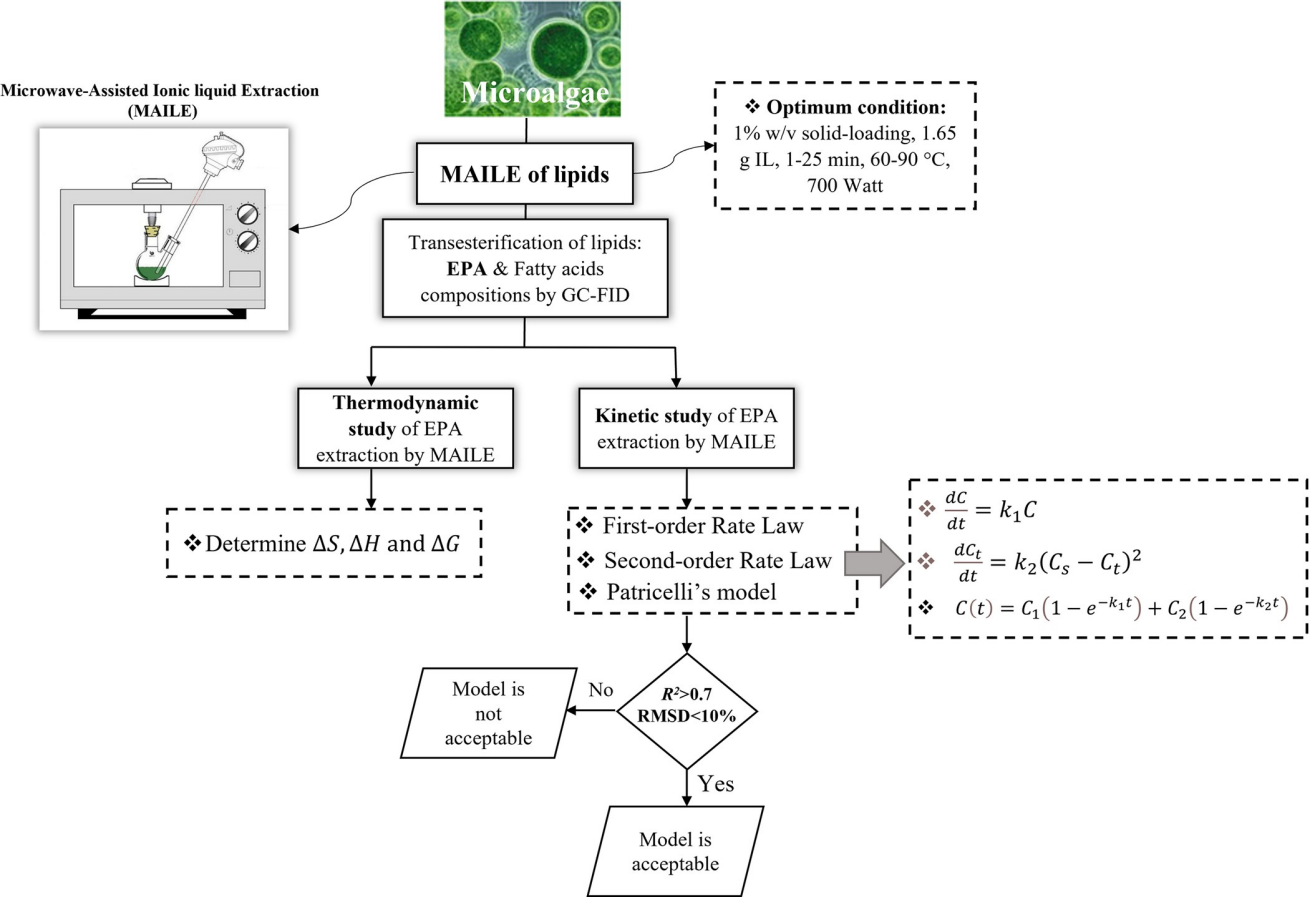
The stepwise development of study models.

In Fig 2, a schema of kinetic and thermodynamic studies of EPA extraction using MAILE is depicted.

### 2.4. Kinetic models for the solid-liquid extraction of EPA

The process of solid-liquid extraction is usually defined by the concentration gradient of the target compounds in the solid phase and the diffusion of each component into the liquid phase. Diffusive mass transfer is modelled via Fick’s second law [30]. This law requires the complex measurement of the concentration gradient inside the cell. At the solid-liquid interface, the diffusive mass transfer is almost equal to the convection mass transfer and the kinetic model [45]. First and second-order kinetic models have frequently been utilized to demonstrate adsorption results attained under non-equilibrium situations. Nevertheless, the kinetics of the extraction process is akin to the adsorption process; thus, the equation applied for adsorption can be employed [34].

The mass transfer of compounds in solid-liquid extraction is comprised of three main steps: a) intra-particle diffusion, which is the diffusion of solid compounds through the porous structure of the solid cell matrix, b) external diffusion that is the movement of a particle throughout the stagnant liquid film within the solid matrix, and c) the release of solid compounds from the solid cell into the liquid medium of the solvent due to thermodynamic partitioning [30]. Considering that the process takes place at a non-steady state without chemical reactions, three different kinetic models: namely first-order and second-order rate laws as well as Patricelli’s model were used to model the extraction of EPA from *N*. *oceanica* using MAILE.

As shown in Eq (4), the first-order rate law was used to evaluate the mass transfer rate of EPA from solid phase into the bulk liquid medium of the solvent [46]:

$$\frac{dC_{t}}{dt}=k_{1}C_{t}$$ (4)

where *k*_*1*_ is the first-order extraction rate constant (min^-1^), and *C*_*t*_ is the concentration of EPA (mg g_FAMEs_^-1^). By integrating Eq (4) at the following boundary conditions, Eq (5) was obtained. At the start of the extraction process (*t* = 0), the initial concentration of EPA is *C*_*0*_. At any time, the concentration of EPS is equivalent to *C*_*t*_:

$$\ln\;C_{t}=k_{1}t+\ln\;C_{0}$$ (5)

where *C*_*t*_ is the integration constant. If the initial amount of extracted EPA is *C*_*0*_, then Eq (5) can be rearranged to evaluate the amount of EPA compounds at any time:

$$C_{t}=C_{0}e^{k_{1}t}$$ (6)

A plot of ln *C*_*t*_ against time at different temperatures generated the slope and intercept of a linear equation. *k*_*1*_ and *C*_*0*_ were obtained through the slope and intercept of Eq (5), respectively.

The second-order rate law of the dissolution of the EPA in the cell matrix of *N*. *oceanica* can be expressed, as in Eq (7):

$$\frac{dC_{t}}{dt}=k_{2}{\left(C_{s}-C_{t}\right)}^{2}$$ (7)

where *k*_*2*_ is the second-order extraction rate constant (min^-1^). *C*_*s*_ is the amount of extracted EPA at saturation in mg g^-1^_FAMEs_ and *C*_*t*_ is the amount of extracted EPA at time *t*. Eqs (9) and (10) can be attained through Eq (8):

$$C_{t}=\frac{C_{s}^{t}k_{2}t}{1+C_{s}k_{2}t}$$ (8)

$$\frac{t}{C_{t}}=\frac{1}{C_{s}^{2}k_{2}}+\frac{t}{C_{s}}$$ (9)

$$\frac{C_{t}}{t}=\frac{1}{1/C_{s}^{2}k_{2}+t/C_{s}}$$ (10)

The initial extraction rate, *h*, as *C*_*t*_*/t* when *t* approaches 0, can be described as:

$$h=C_{s}^{2}k_{2}$$ (11)

Then, EPA concentration at any time (*C*_*t*_) can be obtained using Eq (12). The quantity of *C*_*s*_, *k*_*2*_ and *h* can be experimentally estimated from the slope and intercept by plotting *t/C*_*t*_ against *t*:

$$C_{t}=\frac{t}{1/h+t/C_{s}}$$ (12)

Patricelli’s model of EPA extraction is depicted via a mathematical model developed based on two simultaneous processes, which include a washing stage and a diffusion stage. The first step describes an easy and rapid washing of EPA particles, which are located at the surface of the solid matrix. In the second step, the EPA content diffuses out of the solid cell with the help of the IL solvent mediated in water. Eq (13) describes Patricelli model, which is used to calculate the concentration of EPA extracted *C*_*t*_ at any time (*t*):

$$C_{t}=C_{1}(1-e^{-k_{1}t})+C_{2}(1-e^{-k_{2}t})$$ (13)

where *C*_*t*_ is the EPA concentration (mg g^-1^_FAMEs_) at any given time (*t*). Both *C*_*1*_ and *C*_*2*_ denote the concentration of EPA at equilibrium for the washing and diffusion step (mg g^-1^_FAMEs_), respectively. *k*_*1*_ and *k*_*2*_ are described as the mass transfer coefficients for the washing and diffusion step (min^-1^), respectively. The quantities of *C*_*1*_, *C*_*2*_ as well as *k*_*1*_, *k*_*2*_ were estimated numerically using nonlinear regression. Thus, the total quantity of extracted EPA at equilibrium *C*_*e*_ becomes:

$$C_{e}=C_{1}+C_{2}$$ (14)

The rate of extraction from the first derivative of the Patricelli equation at different extraction temperature can be obtained:

$$h=\frac{dC_{t}}{dt}=C_{1}k_{1}e^{-k_{1}t}+C_{2}k_{2}e^{-k_{2}t}$$ (15)

The extraction rate at the beginning of the reaction can be expressed as:

$$h={\left(\frac{dC_{t}}{dt}\right)}_{t=0}=C_{1}k_{1}+C_{2}k_{2}$$ (16)

Eq (17) is the Arrhenius equation, which demonstrates the correlation between the constants of each kinetic model and the operating temperature:

$$k=Ae^{\left(-\frac{\Delta E_{a}}{RT}\right)}$$ (17)

The rate constant (*k*) is dependent on the temperature based on the Arrhenius relation. *A* is the pre-exponential factor or frequency factor and activation energy (Δ*Ea*) can be found by fitting the experimental data. By taking the logarithm, Eq (17) can be determined:

$$\ln\;k_{1}=\ln\;A-\frac{\Delta E_{a}}{RT}$$ (18)

A plot of ln *k* versus 1/*T*×10^3^ yields a straight line with (−Δ*Ea*/*R*) as the slope, whereby Δ*Ea* and *A* are determined.

### 2.5. Thermodynamic parameters

The thermodynamic parameters of entropy change and enthalpy change for the extraction of EPA from microalgae *N*. *oceanica* using MAILE can be described using Van’t Hoff Eq (19):

$$\ln\;K_{e}=-\frac{\Delta H}{R}(\frac{1}{T})+\frac{\Delta S}{R}$$ (19)

where *K*_*e*_ is the thermodynamic equilibrium constant of the extraction process, *R* is the universal gas constant (8.314 J mol^−1^ K^−1^), and *T* is the temperature used in the process (K). *K*_*e*_ was determined accordingly [47, 48]:

$$K_{e}=\frac{C_{s}}{C_{se}}$$ (20)

where *C*_*s*_ is the concentration of extracted EPA at saturation in mg g^-1^_FAMEs_ and *C*_*se*_ is the concentration of extracted EPA at equilibrium in the biomass. Eq (19) can be rearranged in the form of Eq (21) to incorporate the Gibbs free energy change (Δ*G*):

$$\ln\;K_{e}=-\frac{\Delta G}{R}(\frac{1}{T})=-\frac{\Delta H}{R}(\frac{1}{T})+\frac{\Delta S}{R}$$ (21)

The variations in Gibbs free energy for the different temperatures can be calculated via Eq (22):

ΔG=ΔH−TΔS (22)

where Δ*H*, Δ*S* and Δ*G* refer to enthalpy change (kJ mol^-1^), entropy change (kJ mol^-1^K^-1^) and Gibb’s free energy (kJ mol^-1^), respectively. The plot of ln *K*_*e*_ against 1*/T* produces a straight line while the slope (–Δ*H/R)* refers to the enthalpy and (Δ*S/R)* refers to the entropy change of extraction.

### 2.6. Statistical analysis

The validity of each kinetic model developed for extraction of EPA was estimated by quantifying the differences between the experimental and predicted values using the determination coefficient value (*R*^*2*^). Moreover, the goodness of fit for the kinetic models was assessed using the root mean square deviation (RMSD), and defined as follows:

$$\mathrm{RMSD}=\frac{100}{N}\times \sum \frac{C_{e}-C_{p}}{C_{p}}$$ (23)

where *C*_*e*_ and *C*_*p*_ denote the experimental and anticipated EPA concentration, respectively, and *N* represents the total number of experimental runs. When *R*^*2*^ > 0.7 and RMSD < 10%, the kinetic models are regarded acceptable for the extraction process [19].

## 3. Results and discussion

### 3.1. Kinetics study of EPA using MAILE

**Fig 3:**
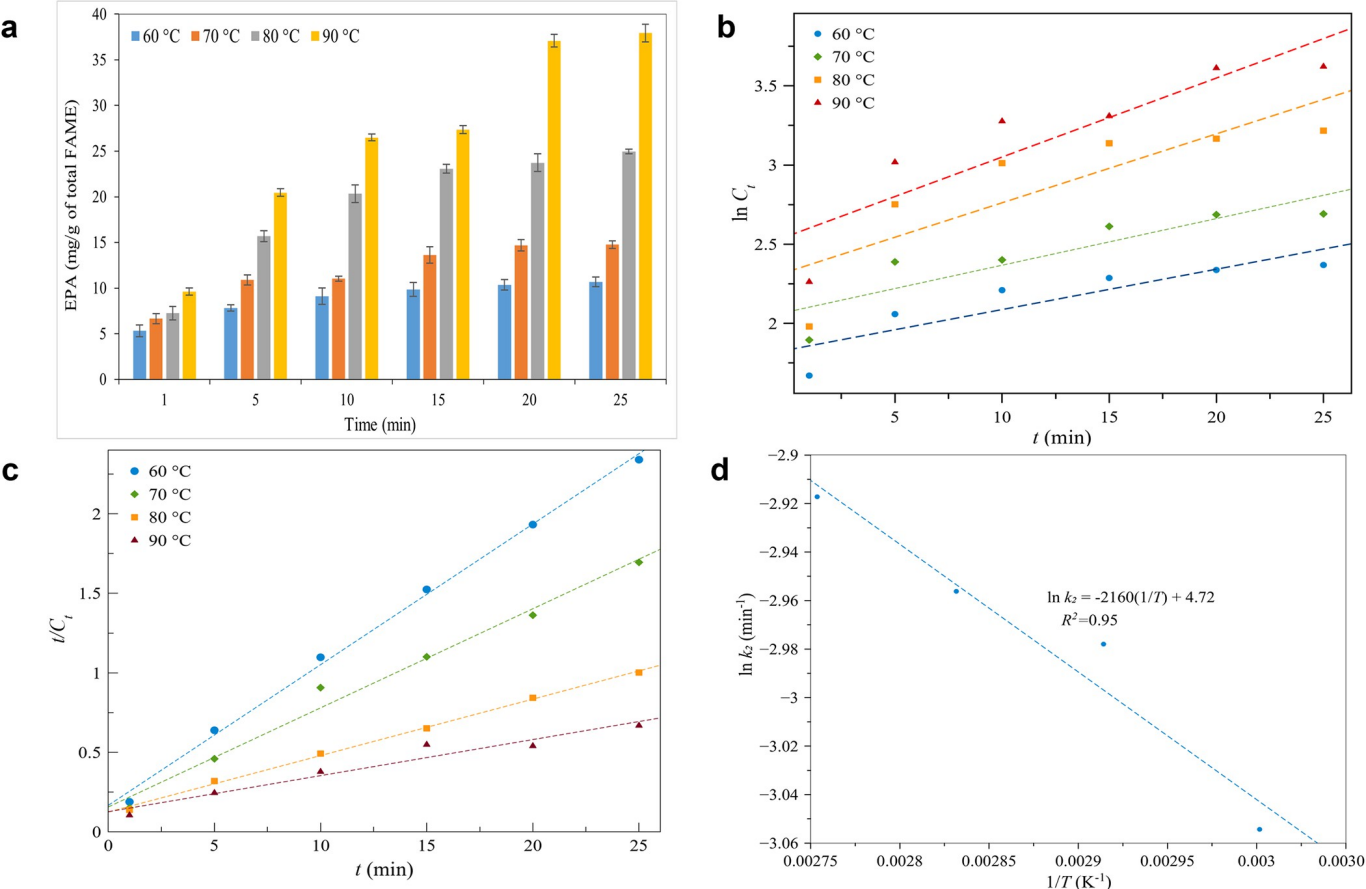
(a) Kinetic extraction of EPA (mg g^-1^_FAMEs_) from *N*. *oceanica* in MAILE using 1.65 g of [TMAm][Cl] at temperature range: 60 to 90°C for 1–25 min, (b) First-order rate law kinetic model of EPA extraction, (c) Second-order rate law kinetic model of EPA extraction, and (d) Relationship between the temperature and second-order rate constant (ln *k*_*2*_). Each experiment was repeated threefold and the values are stated as means ± standard deviations. The error bars represent standard deviation.

After the experimental results were obtained, kinetic analysis was carried out. Results are shown in Fig 3A. Each experiment was repeated three times and values are stated as means ± standard deviations.

When temperature increased by increasing the reaction time, the content of EPA also increased. Maximum EPA content of 37.92 mg g^-1^_FAMEs_ was attained at 90°C after 25 min. According to the temperature applied, the yield of EPA can alter. Such variations in EPA content can be attributed to the solution’s increased solubility and lipids’ decreased viscosity, which promotes the solute’s diffusion into the solution [49]. It is seen that microwave heating had a positive influence on [TMAm][Cl] based extraction. A higher EPA yield infers that the ruptured cells provided accelerated transport of the released EPA compounds into the solvent medium [20].

A similar argument was put forward by [50]. Our findings regarding the influence of reaction temperature and time are consistent with those of a recent work [18], which used microwave-assisted hydrodistillation to extract oils from Sandalwood. Thus, it was found that as the duration of extraction increased, the rate of extraction rose until it hit a plateau or was constant after 120 min. After 1.5 h of extraction, 0.93% extractable oil was collected until it reached the plateau (1.02%). When temperature is increased via MAE, vapor can be improved, and surface tensing decreases, resulting in a decrease in the release of energy from the electromagnetic and microwave radiation. Temperature can increase the diffusivity. This is consistent with Einstein’s equation, which states that increasing the temperature facilitates the increase in diffusivity [51].

According to prior work [42], MAILE was found to be more effective in terms of extraction efficiency and less time-consuming. Our yield was about eightfold that of Soxhlet extraction of EPA (4.83 mg g^-1^_FAMEs_), which required the use of toxic solvents and took six hours.

#### 3.1.1. First-order rate law

The kinetic model based on first-order law was developed. In Fig 3B and Table 1, results are presented. The logarithmic timely concentration (ln *C*_*t*_) against time (*t*) at different temperatures was plotted and indicated that the extraction of EPA can be expressed in linear form. Thus, in Table 1, the kinetic parameters of the first-order model were calculated and depicted.

**Table 1:** First-order kinetic model for the extracted EPA from *N*. *oceanica* microalgae using MAILE.

Extraction temperature (°C)	First-order rate law coefficient *k*_1_ (min^-1^)	Initial concentration *C*_*0*_ (mg g^-1^_FAMEs_)
60	0.026	6.246
70	0.029	7.944
80	0.044	10.236
90	0.049	12.825

In this study, first-order kinetic coefficient (*k*_*1*_) for the extraction of EPA increased ~1.88 times as temperature increased from 60°C to 90°C. This result is consistent with the extraction of essential oil from leaves of *Persicaria minor* utilizing IL based ultrasonication-assisted extraction that found a positive increase in the first-order kinetic coefficient, as temperature increased from 50 to 80°C. In another study, a similar trend was observed: the first-order kinetic coefficient of resveratrol was seen to increase five times, as temperature varied from 16.4 to 83.6°C [52]. Besides, in this study, when temperatures rose, the initial concentration (*C*_*0*_) of EPA accelerated (Table 1).

#### 3.1.2. Second-order rate law

Similar data were analyzed by applying a second-order kinetic model. In Fig 3C, the plot of *t/C*_*t*_ against *t* causes a linear function. Hence, the slope expresses the saturation concentration of EPA (1/*C*_*s*_) and acted as an intercept for the second-order extraction rate constant ($1/C_{s}^{2}k_{2}$), for all examined temperatures.

In Table 2, the effect of temperature on the kinetic parameters deriving from the second-order rate law for the extraction of EPA is displayed. The quantity of *C*_*s*_ was found to increase from 11.299 to 44.053 mg g^-1^_FAMEs_ as the temperature of the solution accelerated from 60 to 90°C. At the higher temperature, weaker interaction among solute–solute and solute–solid caused an increment in *C*_*s*_. Such an increase is in agreement with the study of [53] to extract flavonoids from *Terminalia bellerica* Roxb. using microwave irradiation that found an increase in the saturation concentration of flavonoids from *Terminalia* when the temperature increased from 40 to 100°C.

**Table 2:** Second-order model for the extracted EPA from *N*. *oceanica* microalgae using MAILE.

Extraction temperature (°C)	Second-order rate law coefficient *k*_*2*_ (min^-1^)	Saturation concentration *C*_*s*_ (mg g^-1^)
60	0.047	11.299
70	0.050	16.051
80	0.052	28.169
90	0.054	44.052

To assess the rate of the chemical reaction, *k*_2_ (the second-order rate law) is seen to be vital. Thus, the greater the value of *k*_2,_ the faster the reaction proceeds. As temperature increased, values of *k*_2_ increased. This outcome is probably due to greater thermal energy for solute diffusion. It is evident that the increase in temperature can promote the movement of the solvent into the inner parts as well as enhancing the solubility of the lipid in a solvent. The mechanism of the second-order model implies that EPA extraction happens via two simultaneous processes [22]. At the onset, the extraction of EPA increased rapidly. Later, extraction slowly increased up to completion of the extraction process. Results of the kinetic study demonstrate that the EPA content of lipid products increase sharply during the early stages of the extraction process, but later increase at a slower rate as the process progresses. This outcome is consistent with a study conducted by [22] when the second-order rate law was employed for the extraction of Camptothecin from *Nothapodytes nimmoniana* plant via MAE. Then, as extraction temperature increased from 40 to 60°C, the *k* values varied from 0.026 to 0.035 min^-1^.

In theory, the solvent used for extraction diffuses into the solid matrix and reaches the cell core where EPA is present. Thus, EPA dissolves into the solvent until its concentration limit is reached. The solution, which contains the dissolved EPA diffuses back to the solid surface and transfers into the bulk solution via convection [54].

In Fig 3D, both activation energy and frequency factor were measured by plotting ln *k*_*2*_ versus *1/T*. From the slope and intercept of the straight line, activation energy and frequency factor for the extracted EPA attained 17.958 kJ mol^-1^ and 1.12×10^2^ min^-1^, respectively. When the value of activation energy is lower than 20 kJ mol^-1^, the extraction process is governed by the diffusion [55]. Thus, the solid–liquid extraction of EPA from microalgae via the second-order kinetic model reveals that the reaction rate is diffusion-controlled [52]. Microwave energy is believed to enhance the diffusional process by accelerating the solid particle permeability of the solvent, thereby releasing EPA. It can also be claimed that the lower energy restriction necessary to begin diffusion is aided by microwave energy, which may help to overcome the solute-solute and solute-matrix interactions, so diminishing the Δ*E*_*a*_ of the extraction process [56].

Our findings are consistent with previous literature whereby activation energy for the extraction of flavonoids from *Terminalia bellerica* with assistance of microwave irradiation of 12.07 kJ mol^-1^ reached temperatures between 40–100°C with a solvent-to-solid ratio of 40 ml g^-1^ [53]. Another study reported that quantities of activation energy and frequency factor were found to be 17.74 J mol^-1^ and 2.21 min^-1^ respectively for the production of biodiesel from palm fatty acid distillate [57]. Next, another study into activation energy and the Arrhenius constant of microwave-assisted extraction of Aegle Marmelos Correa (AMC) oil attained 16.428 kJ mol^-1^ and 344.4 s^-1^ [58].

#### 3.1.3. Patricelli kinetic model

**Fig 4:**
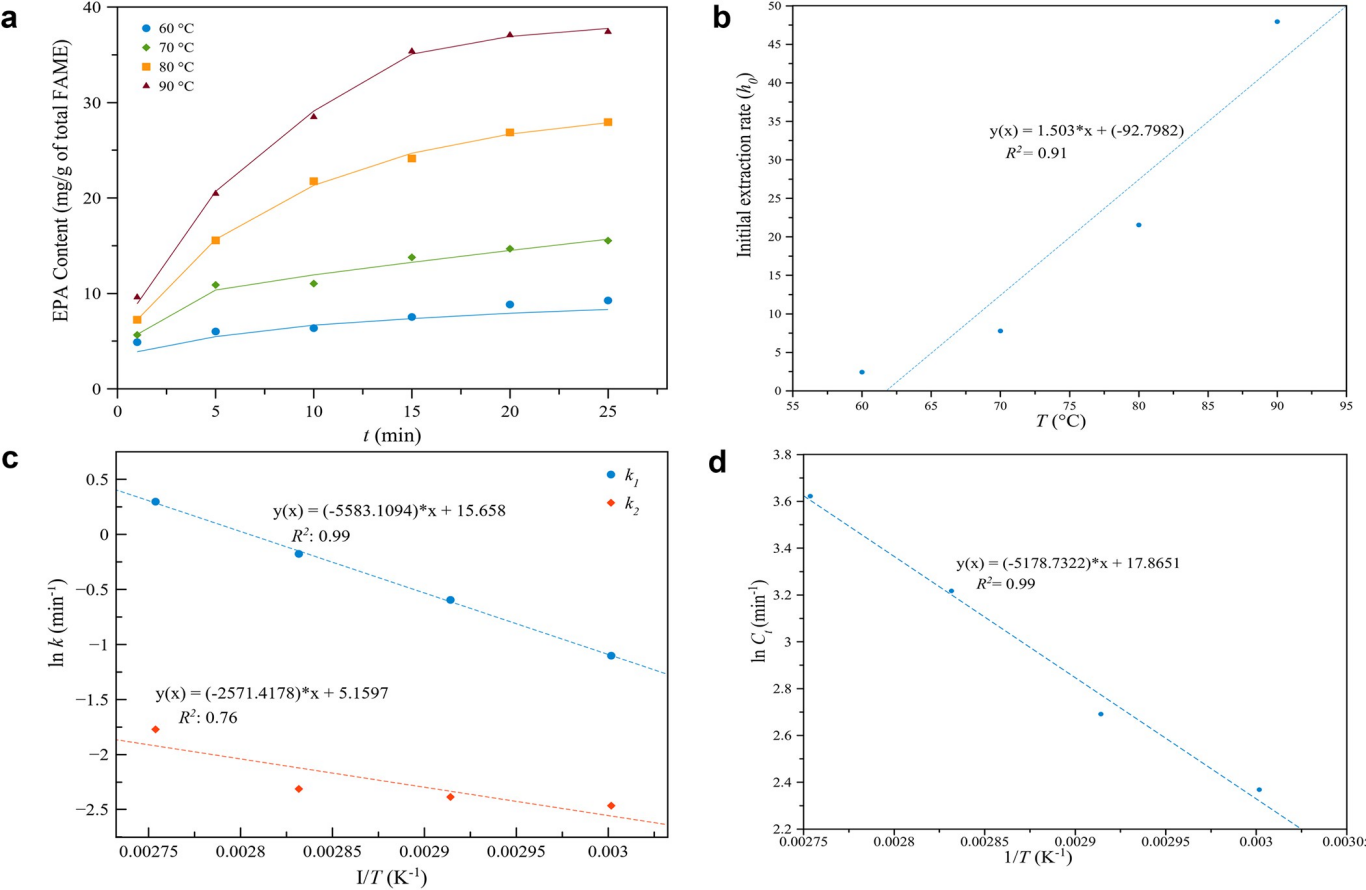
(a) Kinetic profiles of extracted EPA (mg g^-1^_FAMEs_) from *N*. *oceanica* in MAILE using 1.65 g of [TMAm][Cl] temperature range: 60 to 90°C for 1–25 min fitted by Patricelli’s model. Symbols: experimental data; lines: model fitting curves, (b) Relationship between temperature and Patricelli’s model initial extraction rate of EPA, (c) Relationship between the temperature and Patricelli’s model mass transfer coefficient at washing (*k*_*1*_) and diffusion (*k*_*2*_) phase and (d) Determination of *ΔH* values from the plot of ln *C*_*t*_ against 1/T (K^−1^).

The empirical kinetic model developed by Patricelli’s model was studied to fit the EPA extraction. In Eq 13, using Polymath software to fit Patricelli’s model, it was seen that the experimental data regressed. In Fig 4A, the profiles of experimental and calculated values of the extracted EPA at various levels of temperatures via MAILE technique are shown.

In Fig 4A, as temperature increased from 60 to 90°C, extraction of EPA increased. Extraction of EPA quickly intensified throughout the washing stage at the start of the process from 1 min to 15 min. As for the diffusion stage, EPA extraction increased gradually lasting over 15 min. The washing stage was credited to be more prominent than the diffusion stage since the values of extracted EPA placed on the outer surface of the microalgae cells were higher than the values located inside the cells of the microalgae [37]. In Table 3, the corresponding values of Patricelli’s model, which were obtained from the experimental data for temperature variation, are displayed.

**Table 3:** The Patricelli kinetic model for the extracted EPA from *N*. *oceanica* microalgae using MAILE.

	Mass transfer coefficients (min^-1^)		Equilibrium concentration (mg g^-1^)		
Extraction temperature (°C)	*k*_*1*_	*k*_*2*_	*C*_*1*_	*C*_*2*_	*C*_*e*_
60	0.332	0.085	8.469	1.890	10.359
70	0.551	0.092	13.190	1.974	15.164
80	0.837	0.099	22.765	2.613	25.378
90	1.345	0.170	35.316	2.659	37.975

In Table 3, the mass transfer coefficients (*k*_*1*_ and *k*_*2*_) for the two phases increased as temperature increased. The coefficients of EPA at the washing step proved to be higher than at the diffusion step (*k*_*1*_ > *k*_*2*_) for all tested temperatures. Hence, the highest value of *k*_*1*_ of 1.345 min^-1^ at 90°C was obtained for the extracted EPA. In Fig 4A, the rapid extraction rate at the outset, known as the washing stage, enabled the fast dissolution of target components i.e. EPA both at the surface and within the broken matrix cells. In contrast, the diffusion stage was more gradual because of mass transfer restrictions; the left active compounds diffused from the inside of intact cells into the solvent [37].

Further, it is noted that for the washing stage (*C*_*1*_), the yield of EPA was higher than that of the diffusion stage (*C*_*2*_) for all microwave temperature variations, demonstrating that nearly all EPAs were extracted throughout the washing stage (*C*_*1*_ > *C*_*2*_). This outcome arose because EPA from the outer surface of the microalgae can readily dissolve into the extracting solvent due to microwave irradiation. Towards the end, the EPA diffuse from the intact microalgae cells inside the solvent, and thereby extraction rate is reduced [19].

In Table 3, based on the EPA values at equilibrium for the washing and diffusion phases, the values of *C*_*e*_ at each extraction temperature were calculated. In Fig 4A, the extracted microalgae EPA are seen to increase rapidly at the start of the extraction process, as determined using Eq (13); EPA extraction decreased quickly afterward. To verify this trend, the calculations of the extraction rate (*h*), according to Eq (15) are shown in Table 4. Findings show considerable enhancement in the extraction rate of EPA content at the start of the extraction reaction. However, immediately afterwards, the extraction rate of EPA content reduced. In Fig 4B, it can be seen that the initial rate at the very initial extraction rate (*h*_*0*_) increased along with the reaction temperature. The initial rate increased linearly, attaining a high coefficient of 0.91 for the extracted EPA. The initial rate at 90°C extraction temperature was 19 times higher than that of 60°C. Such an outcome highlights the effectiveness of using MAE for extraction at higher temperatures. This finding is in accordance with the extraction of total lipids from *Nannochloropsis sp*. using microwave-assisted NaCl [19]; where it was reported that the initial extraction rate at 100°C extraction temperature was six times higher than that of 60°C. These results confirm the higher extraction efficiency of microwave used at higher temperature.

**Table 4:** The variation of extraction rate of EPA against reaction time using MAILE.

Time (min)	Extraction rate (min^-1^)			
	60°C	70°C	80°C	90°C
0	2.432	7.781	21.543	47.943
1	1.777	4.545	9.451	12.754
5	0.537	0.598	0.482	0.249
10	0.151	0.103	0.101	0.082
15	0.061	0.048	0.059	0.035
20	0.032	0.029	0.036	0.015
25	0.020	0.018	0.021	0.001

In Fig 4C, the Arrhenius plots for the extracted EPA are shown, for both the washing and diffusion phase. The high determination coefficient values of 0.99 and 0.76 were achieved by plotting ln *k*_*1*_ and ln *k*_*2*_ versus 1/*T*. The estimated activation energy of EPA extraction for the washing phase was around 46.42 kJ mol^-1^ and the activation energy for diffusion phase was 21.38 kJ mol^-1^, in the temperature range of 60–90°C. The Patricelli kinetic model was used to determine the minimum energy essential to extract EPA from microalgae *N*. *oceanica* and was found to be 67.8 kJ mol^-1^.

It is evident that the generation of products occurs only when the energy of the reactant is equal or higher than the activation energy. The activation energy obtained for EPA production from microalgae biomass in this research is greater than the activation energy found for microwave irradiation of lipids generated from *Nannochloropsis sp*. microalgae utilizing NaCl (50.5 kJ mol^-1^) [19]. A biomass having a different cellular matrix membrane structure can absorb a distinct level of permeability and complexity, presenting a different activation energy while in extraction. The presence of unsaturated compounds can result in slower reaction rates as well as higher the activation energy [45]. In a study of palm oil non-catalytic transesterification, it was found that the activation energy of a specific reaction highly depends on the reaction parameters such as temperature, catalyst loading, and the nature of the reactants [59]. This fact was also approved in the study of biodiesel production from *S*. *platensis* algae wherein the activation energy depends on the catalyst used [45].

In Fig 4D, the pre-exponential factors for EPA extraction were found to be 6.31×10^6^ min^-1^ for the washing phase and 1.74×10^2^ min^-1^ for the diffusion phase. The values of the pre-exponential factors proved to be higher for the washing stage compared with the diffusion stage. This shows that the frequency of collision of two or more molecules during the washing phase is higher compared to the diffusion phase [53].

#### 3.1.4. Comparison of the kinetic models

Regardless of the values of the model parameters or their similar behavior, *R*^*2*^ and RMSD values were used to determine the best models that fit the experimental data for the linear kinetic models. The greater the *R*^*2*^ value and the smaller the RMSD value, the more accurately the model fits the experimental data [39]. In Table 5, the values of *R*^*2*^ and RMSD across various reaction temperatures, obtained for first-order and second-order rate laws together with Patricelli’s models, are presented.

**Table 5:** Statistical parameters of the kinetic modelling for MAILE of extracted EPA.

Extraction temperature (°C)	First-order rate law		Second-order rate law		Patricelli’s model	
	*R* ^*2*^	*RMSD (%)*	*R* ^*2*^	*RMSD (%)*	*R* ^*2*^	*RMSD (%)*
60	0.641	21.037	0.997	7.915	0.971	7.441
70	0.788	20.042	0.986	7.168	0.967	8.735
80	0.601	20.531	0.998	7.062	0.952	8.973
90	0.705	19.217	0.959	9.821	0.980	6.975
**Average**	0.684	20.207	0.985	7.992	0.966	8.031

According to the results, the RMDS percentages for both the second-order and Patricelli’s models were less than 10%. However, for the first-order law, the RMSD percentage was found to be greater than 10%. The average *R*^*2*^ value for first-order rate law was also less than 0.7 whereas the average *R*^*2*^ value for second-order rate law and Patricelli’s models were higher than 0.7. Thus, this outcome depicts a better correlation of the second-order rate law with the experimental results than Patricelli’s model. The first-order rate law model failed to describe the kinetic behavior of EPA extraction using MAILE.

These findings corroborate a prior study conducted by [18]. The author discovered that, in comparison to the first-order extraction model, the second-order model had extremely high coefficients of determination, which may explain the extraction of oils from Sandalwood using microwave-assisted hydrodistillation. The second-order rate law model has successfully explained the kinetics of essential oil extraction from patchouli leaves [60] Sandalwood [18] and almonds of Syagrus cearensis [61] using non-conventional methods such as MAE, microwave air-hydrodistillation method, and ultrasonic, respectively. In addition, another study [62] demonstrated that a second-order kinetic model for essential oil extraction procedures from *C*. *camphora* had a high *R*^*2*^ (0.9996) for solvent-free microwave assisted extraction (SFME) and 0.9997 for conventional hydrodistillation (HD), indicating that such a model is capable of accurately representing the determined results for the two essential oil extraction processes.

### 3.2. Thermodynamic study of MAILE of EPA

**Fig 5:**
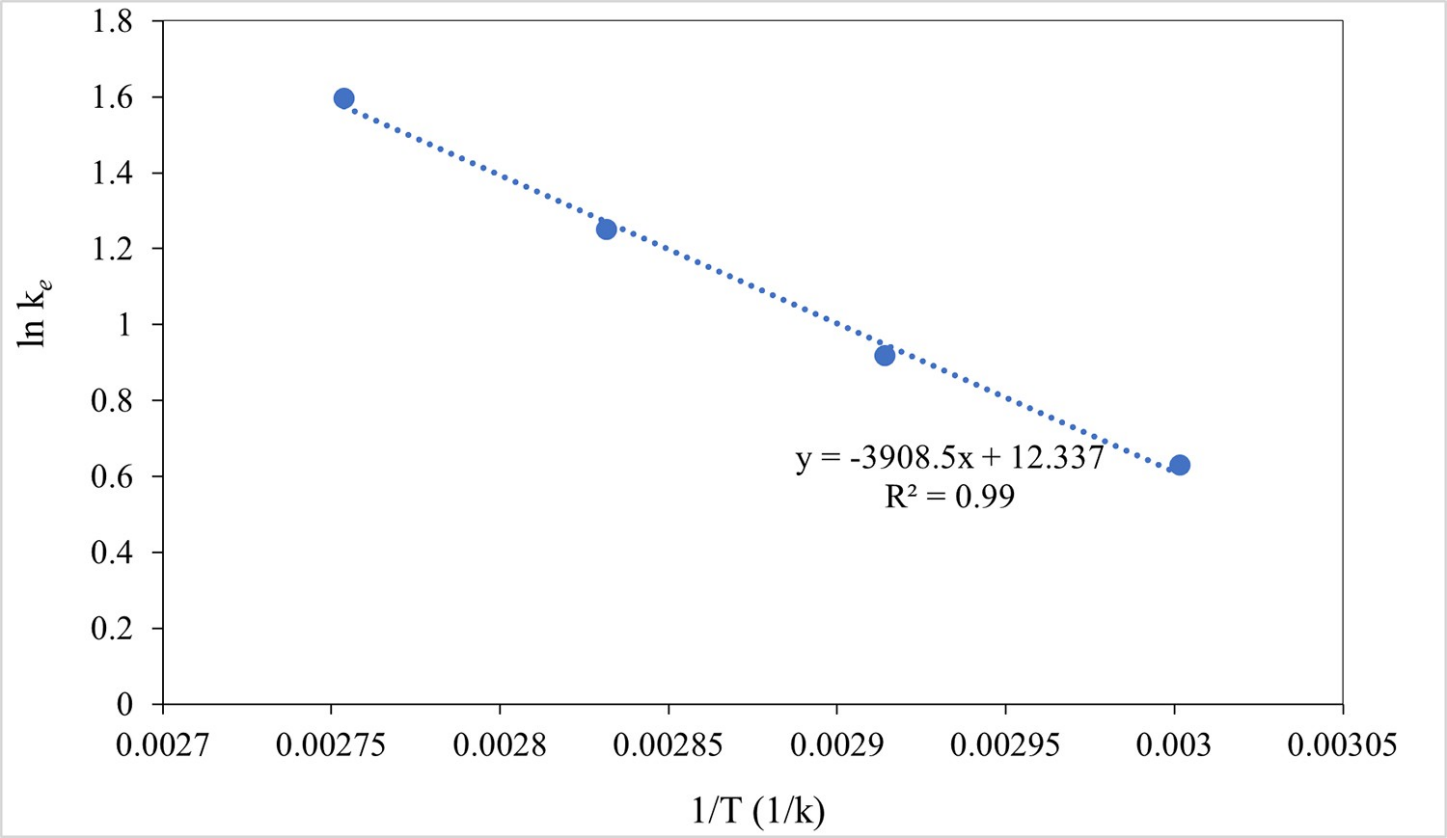
Plot of ln *K*_*e*_ against 1/T for EPA extraction process to determine the thermodynamic parameters.

In Table 6, the thermodynamic parameters for EPA extraction employing MAILE are shown. In Fig 5, graphs of ln *K*_*e*_ against 1/*T* for varying reaction temperatures from 60 to 90°C are displayed.

**Table 6:** Thermodynamic parameters (Δ*H*, Δ*S* and Δ*G*) for the extraction of EPA from *N*. *oceanica* at different temperatures.

Temperature (°C)	Temperature (K)	*K*_*e*_	Δ*H* (kJ mol^-1^)	Δ*S* (kJ mol^-1^K^-1^)	Δ*G* (kJ mol^-1^)
60	333.15	1.88	32.50	0.10	-1.68
70	343.15	2.50			-2.70
80	353.15	3.50			-3.73
90	363.15	4.94			-4.75

The equilibrium constant (*K*_*e*_) values were determined using Eq (19). When temperature rises, *K*_*e*_ increases. Such an outcome is consistent with prior work [47] on the MAE-mediated extraction of paclitaxel from Taxus chinensis. The thermodynamics of EPA extraction revealed an enthalpy value of 32.50 kJ mol^-1^ at temperatures ranging from 60 to 90°C. Such data demonstrate that the *ΔH* values were positive, which indicates the extraction process of EPA is endothermic (heat-absorbing) and needs more energy. Henceforth, the extraction process absorbs an external energy necessary for efficient extraction. On the other hand, heat input is vital to bring the reactants to the transition state in order to form products. The positive enthalpy change agrees relatively well with other studies on the extraction of Jatropha oil [63], cottonseed oil [64], and Colocynthis vulgaris Shrad (melon) seeds oil [39].

*ΔS* signifies whether the system can be returned to the initial state or not after the process is complete. Here, the positive value of *ΔS* (0.10 kJ mol^-1^K^-1^) shows that the process is irreversible and that the reaction’s spontaneity is more profitable; thereby in agreement with the results of [29, 54]. The values of *ΔG* proved to be -4.75 kJ mol^-1^ at 90°C. Free Gibbs energy determined the energy profile of the system. An increase in the temperature of the reaction initiates a spontaneous reaction that can proceed. The negative value of Gibbs free energy shows that the extraction process is favorable at higher temperatures. This outcome is consistent with previous studies of oil extraction from Colocynthis vulgaris Shrad (melon) seeds [39] and Irvingia gabonensis kernel [29]. The thermodynamic properties found in this research such as irreversibility, the spontaneous and endothermic nature of extracted EPA are in agreement with previous studies such as the extraction of oil from olive cake [65]; oil extraction from *Jatropha curcas* L. [66]; and oil extraction from white mustard [67].

There are a number of technical and technology-specific limitations in performing MAILE for this study. For instance, the quality of extraction could not be determined at temperatures above 100°C due to water evaporation. Thus, optimizing such systems requires a deeper knowledge of the underlying chemical reaction mechanisms in order to achieve high extraction efficiency even at low temperatures. Furthermore, since the study employed batch extraction, some difficulties were found in thermodynamically stabilizing the system, as it needed certain additional instruments and pretreatments.

Regarding the positive outcome of this study, scaling up the process must be considered in future research by evaluating capital costs, energy requirements, and economics associated with industrializing the MAILE process. Moreover, additional research is necessary to fully understand the mechanism of MAILE as a continuous process in order to build an eco-friendly system.

## 4. Conclusions

In this paper, the kinetics of the extracted EPA from *Nannochloropsis oceanica* microalgae using [TMAm][Cl] based MAE was determined. It was found that the second-order rate law best fitted the experimental data. Statistical findings indicated the greatest average values of *R*^*2*^ (0.986) and the lowest average values of RMSD (7.992). Thermodynamic parameters confirmed that the process of extraction is endothermic in nature, irreversible and spontaneous. In addition, results highlight the pivotal role of temperature and time in EPA extraction. Results also confirmed the potential of MAILE process for the extraction of high-quality EPAs for both pharmaceutical and nutraceutical applications.

## Supporting information

S1 Data (XLSX)
